# Evidence for the Necessity of Objective Hearing Tests in Cochlear Implantation Assessment: Excluding Functional Hearing Loss Cases

**DOI:** 10.3390/jcm14103585

**Published:** 2025-05-20

**Authors:** Anita Gáborján, Márton Kondé, Marianna Küstel, Nóra Kecskeméti, László Tamás, Ildikó Baranyi, Gábor Polony, Judit F. Szigeti

**Affiliations:** 1 Department of Otorhinolaryngology, Head and Neck Surgery, Semmelweis University, 1083 Budapest, Hungary; konde.marton.vilmos@semmelweis.hu (M.K.); kustel.mariann@semmelweis.hu (M.K.); kecskemeti.nora@semmelweis.hu (N.K.); tamas.laszlo@semmelweis.hu (L.T.); baranyi.ildiko@semmelweis.hu (I.B.); polony.gabor@semmelweis.hu (G.P.); szigetifjudit@semmelweis.hu (J.F.S.); 2 Department of Voice, Speech and Swallowing Therapy, Faculty of Health Sciences, Semmelweis University, 1085 Budapest, Hungary

**Keywords:** cochlear implant, functional hearing loss, assessment, ASSR, ABR, risk factors, rehabilitation

## Abstract

**Background/Objectives**: Cochlear implantation is a crucial intervention for individuals with severe hearing loss, aiming to restore auditory function and improve quality of life. The decision to recommend cochlear implantation critically depends on accurate audiological evaluations. However, challenges arise when subjective assessments of hearing loss do not align with objective audiological measurements, leading to potential misdiagnoses. Comparisons are to be made between subjective and objective results, with an investigation into the characteristics, warning signs, and risk factors of functional hearing loss (FHL). **Methods**: A retrospective study of hearing loss presentations at an otorhinolaryngological university clinic between 2020 and 2024 was performed, whereby we collected FHL cases. The evaluation process included measurements of subjectively perceived hearing loss through pure-tone audiometry, speech understanding, and communication testing. The objective assessments comprised impedance measurement, otoacoustic emission measurement, auditory brainstem responses, auditory steady-state responses, and medical imaging. **Results**: During the studied period, 11 patients, with an average age of 35.2 years (13 to 64 years), who were originally referred for cochlear implantation evaluation and subsequently diagnosed with FHL, were identified. The majority (10 patients) were female. No organic cause was identified in four cases, while seven cases exhibited some organic ear abnormalities insufficient to justify the reported hearing loss. The degree of FHL ranged from 30 dB to 90 dB, with an average of 60 dB. **Conclusions**: Diagnosing FHL is challenging and requires comprehensive assessment and interdisciplinary collaboration. Failure to recognize it may lead to inappropriate treatment, including unnecessary cochlear implantation. This study advocates for the mandatory integration of ABR and ASSR in the clinical evaluation of all cochlear implant candidates to ensure accurate diagnosis and optimal treatment.

## 1. Introduction

In the rehabilitation of severe hearing loss, cochlear implantation (CI) offers a solution that can improve patients’ hearing, speech perception, and communication skills. CI has undergone a significant expansion in its indications in recent years [1,2,3,4]. In 1984, at the time of receiving approval from the U.S. Food and Drug Administration (FDA), the candidacy criteria for CI were restricted to individuals with profound bilateral hearing loss. However, it is now increasingly recognized as a viable rehabilitative option even for individuals with lesser degrees of hearing loss who do not achieve sufficient benefit from conventional hearing aids, particularly in cases of poor aided speech perception. Therefore, its candidacy now includes younger recipients as well as individuals with greater residual hearing and more advanced speech perception abilities [5,6]. In line with this shift, the candidacy criteria have also broadened to include individuals with asymmetric or unilateral sensorineural hearing loss [7,8,9]. Clinical experiences further suggest that the indication criteria can be expanded to include patients with an unaided pure-tone average of 60 dB HL or greater and an unaided word recognition score of 60% or less in one or both ears [3]. 

The evaluation process in CI centers has become more standardized, with routine assessments forming the basis of candidate selection. Despite this, individualized consideration remains a critical factor in optimizing rehabilitation outcomes. A multidisciplinary implantation team is responsible for conducting the preoperative evaluation, performing the surgery, and overseeing postoperative rehabilitation. The assessment process includes mandatory examinations determined by the implant team as well as additional evaluations tailored to the individual needs of the candidate. While most patients present with hearing complaints that clearly justify CI, there are occasional cases in which the clinical presentation does not align with the audiological findings. Functional hearing loss represents one such diagnostic challenge.

Several terms are in use denoting the phenomenon described in the present paper, e.g., ‘conversion deafness’, ‘psychogenic hearing loss’, or ‘hysterical hearing loss’ as older terms, and ‘pseudohypacusis’, ‘nonorganic hearing loss’, or ‘functional hearing loss’ as newer, often synonymous terms [10,11]. In this article, we chose to adopt ‘functional hearing loss’ (FHL) as one of the most neutral terms, with the fewest negative connotations, and the least reference to etiological background or possible intent. We define FHL as false or exaggerated hearing loss evident on audiological testing, but with no corresponding organic impairment, or more severe than can be explained by the pathology identified in the auditory system [12,13,14]. This category overlaps with the psychiatric diagnosis ‘functional neurological symptom disorder’, also referred to as ‘conversion disorder’, a somatic disorder that presents with one or more neurological symptoms that do not clinically correlate with recognized neurological or medical conditions, and brought on by intense stress, emotions, or an associated psychiatric disorder [15]. Conversion disorders are rare, documented in fewer than 1% of individuals in community settings [16], with motor problems being among the most frequent manifestations, and FHL among the most sporadic [17]. The risk factors for FHL include female gender, young age, prior hearing difficulties, exposure to others with similar symptoms, significant stress, and premorbid psychological conditions such as pre-existing conversion and major depressive or anxiety disorders [18,19,20].

Hearing threshold assessment through pure-tone audiometry and speech audiometry often provides clear indications for CI. Subjective audiometric methods yield highly precise results, with a margin of error of approximately 5 dB. Objective diagnostic techniques are mainly used with pediatric patients, where they play a crucial role in determining objective hearing thresholds, as the reliability and feasibility of subjective methods are limited in this population. Adult patients, however, usually offer hearing threshold information consistent with measurable data; therefore, objective techniques are not customary with this population [21,22,23]. When applied, objective examinations, such as the auditory brainstem response (ABR) and auditory steady-state response (ASSR), are predominantly used for differential diagnostic purposes. Additionally, otoacoustic emission (OAE) testing provides valuable information regarding inner ear function. This article aims to offer arguments for the necessity of objective hearing assessments in differentiating FHL from objectifiable sensorineural deficits, thereby supporting clinical decision-making in CI in all ages.

## 2. Materials and Methods

### 2.1. Patients

Patients referred to the Department of Otorhinolaryngology, Head and Neck Surgery of Semmelweis University, Budapest, with a CI indication were examined between July, 2020, and July, 2024 (a 4-year period). The inclusion criteria were the following: better-than-expected communication skills and/or objectively measured hearing thresholds lower than subjectively reported ones, and a primary indication for CI, based on either clinical evaluation or external referral with a diagnosis of severe-to-profound hearing loss. The exact methodology of measurement and difference calculations is detailed in Section 2.5. The exclusion criterion was potential malingering in individuals for whom a secondary gain from the hearing loss diagnosis could not be ruled out. That is, we did not directly assess for deception, but excluded all cases where even a minimal suspicion of malingering arose, e.g., if the primary motivation appeared to be benefit-oriented (e.g., obtaining disability support or financial gain, forensic cases) and objective assessments yielded better hearing thresholds than those subjectively reported. Such individuals were not classified as cases of FHL. The ages of the patients were recorded based on their first visit to the clinic. All participants were assessed according to the same protocol, with identical inclusion criteria and testing procedures applied irrespective of gender, in order to minimize the risk of gender-based bias. In all cases, patient history was taken through both direct patient anamnesis and heteroanamnesis, followed by a physical examination. Revealing tests are valuable tools in diagnosing FHL. These tests are meant to reveal discrepancies in patients’ responses to unexpected stimuli or questions, making it difficult for individuals with FHL to maintain a consistent, “false” hearing profile. In spontaneous interactions in which an unpredictable event, such as a sudden noise deliberately produced behind the back of the patient, occurs, or when an utterance unrelated to the given situation is produced, such as ‘Has it started raining?’, the patient’s natural reactions and behavioral changes can either confirm or refute the presence of severe hearing impairment, providing insight into their actual hearing abilities.

### 2.2. Subjective Audiological Methods

Hearing status was assessed through speech-based evaluation, based on comprehension (understanding questions, following requests and instructions) and production (volume, articulation, intonation, and responses to questions). Pure-tone audiometry was conducted to measure air conduction hearing thresholds at 125, 250, 500, 1000, 2000, 4000, and 8000 Hz, while bone conduction thresholds were assessed at 250, 500, 1000, 2000, and 4000 Hz using a GSI 61 clinical audiometer. Speech recognition tests were performed according to protocols suggested by Árpád Götze’s monosyllabic speech definition test in Hungarian. Additionally, an evaluation was performed by a deaf and hard-of-hearing teacher involving the observation and an analysis of spontaneous reactions, as well as verbal and non-verbal responses to targeted questions and tasks. As a so-called ‘revealing test’, patients’ reactions to unexpected questions were observed.

In our subjective observations, we examined patients’ motivation to communicate, conversational engagement, speech comprehension, verbal expressions, and response quality, and the lack or presence of characteristics typically associated with severe hearing impairment. The assessment of vocabulary, speech intelligibility and pronunciation, grammaticality, and suprasegmental aspects of speech, such as prosodic elements, serves as an important reference point for evaluating the actual severity of hearing loss [24].

### 2.3. Objective Audiological Methods

Impedance audiometry was performed, including tympanometry and stapedius reflex examination. Otoacoustic emission measurement was conducted using a distortion product otoacoustic emission (DPOAE) test at 12 frequencies between 500 and 10,000 Hz with an Interacoustics Titan device. Auditory brainstem response (ABR) testing was performed using an Interacoustics Eclipse device with click stimulus application. Objective hearing threshold assessment was conducted with air conduction through auditory steady-state responses (ASSRs) at 500, 1000, 2000, and 4000 Hz using an Interacoustics Eclipse device.

### 2.4. Imaging

Comprehensive brain and targeted inner ear MRI scans were performed with a contrast agent to visualize or rule out possible central structural abnormalities. In all cases, HRCT or PCCT of the temporal bone was also conducted.

### 2.5. Data Processing

**Figure 1:**
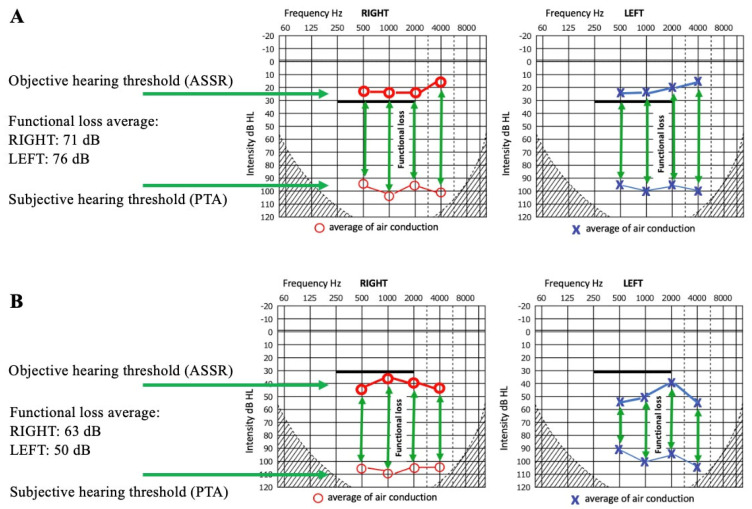
The average of the subjective and objective hearing thresholds at 500, 1000, 2000, and 4000 Hz in patients diagnosed with FHL. The discrepancy between the two measurements is represented as FHL. (**A**)—Cases with normal hearing (4 patients, 8 ears). (**B**)—Cases with some degree of organic ear disease (7 patients, 6 right ears, 5 left ears).

The degree of FHL was calculated based on the difference between subjective and objective hearing thresholds measured at speech frequencies (Figure 1). We compared the hearing thresholds obtained by air conduction at 500, 1000, 2000, and 4000 Hz during pure-tone audiometry with the hearing thresholds determined objectively by ASSR measurements at 500, 1000, 2000, and 4000 Hz. The values measured at the four frequencies (500, 1000, 2000, and 4000 Hz) were averaged for the right and left ears separately. The mean subjective (PTA) and objective (ASSR) thresholds and differences in the degree of FHL are presented in Table 1. Based on the findings in the literature and our clinical experience, the mean differences between ASSR and PTA thresholds are typically within clinically acceptable limits—generally 4–10 dB across key frequencies. Several studies have demonstrated a strong correlation between ASSR and PTA thresholds, with correlation coefficients ranging from r = 0.89 to 0.96 across frequencies, confirming a reliable linear relationship. Moreover, over 85% of threshold differences fall within 20 dB in both normal-hearing and hearing-impaired individuals [25,26,27]. Therefore, we considered a discrepancy between subjective and objective measurement results to be significant if it exceeded 20 dB. In several cases, this functional loss did not account for the total hearing impairment, as some patients exhibited a combination of organic and functional hearing loss, which contributed to the indication for CI together. All patients were presumed to be CI candidates based on subjective measurements (see Table 1, PTA results).

Risk factor analysis was also conducted by the psychologist.

The retrospective analysis and retrieval of data from patient documentation were conducted with the approval of the Regional and Institutional Scientific and Research Ethics Committee (reference number: SE RKEB 216/2022; date: 16 November 2022).

## 3. Results

During the investigated 4 years, there were 209 cochlear implant candidates over the age of 6 at the clinic. This age threshold was chosen because below this age, assessment necessarily relies on objective diagnostic methods. Among the CI candidates, 11 patients (19 ears) exhibited better performance in objective tests compared to subjective assessments. A clear female predominance was noted, with 10 female and only 1 male patient. In terms of age, the patients ranged from 13 to 64 years, with a mean age of 35.2 years (Table 1).

The average of the functional hearing impairment was 60 dB. Intact hearing was confirmed by objective hearing tests in eight ears of four patients. In these cases, the average FHL was 71 dB in the right ear and 76 dB in the left ear. In 11 ears of seven patients, a functional component was present in addition to some degree of organic hearing loss. Regarding the difference between perceived and actual hearing loss, the average FHL was 63 dB in the right ear and 50 dB in the left ear (Figure 1).

For Figure 1, the patients’ hearing thresholds were averaged at each individual frequency. For averaging, only the affected ear was considered in unilateral cases. If the patient did not indicate a hearing threshold, deafness was assumed, and a hearing threshold of 120 dB was used. The objective hearing threshold measured with ASSR, even in cases of normal hearing, rarely reaches 0 dB. Due to technical limitations (electrical background noise, brainwave activity), it is difficult to measure 0 dB at low sound intensities, especially at lower frequencies (500, 1000 Hz), so an objective threshold of 10–20 dB is considered within the normal hearing range. Considering these factors (a maximum subjective threshold of 120 dB and an objective threshold of 10–20 dB instead of 0 dB), the calculated differences (representing functional loss) may be smaller than in reality. Therefore, the obtained average value represents the minimal degree of FHL, which is likely slightly higher in reality. As we compared air-conduction thresholds obtained through PTA and ASSR, both of which depend on sound transmission through the middle ear, even in cases of conductive hearing loss, air-conduction thresholds obtained via ASSR closely correlate with those from PTA. Both methods are similarly affected by middle ear pathology [28,29]. Therefore, in our cohort, discrepancies between the two methods cannot be attributed to middle ear pathology, even in cases of conductive hearing loss.

Among the 11 participants included in the study, two individuals demonstrated fully adequate communication based on the communicative engagement–conversational involvement scale. Two participants exhibited a complete lack of communication. The remaining seven patients displayed varying levels of communication abilities between these two endpoints.

**Figure 2:**
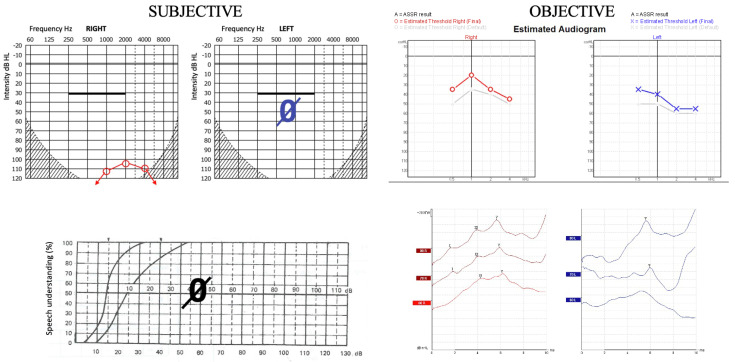
A comparison of subjective and objective audiological assessments in a case presentation (P1). Pure-tone audiometry and speech audiometry indicate residual hearing on the **right** side and deafness on the **left** side with 0% speech recognition. ASSR and ABR testing reveal mild hearing loss on the **right** side and moderate hearing loss on the **left** side.

In one illustrative case (P1), a significant discrepancy was observed between the subjective and objective audiological test results. Pure-tone audiometry and speech audiometry indicated residual hearing on the right side and profound hearing loss on the left side, with 0% bilateral speech recognition. In contrast, objective assessments including ASSR and ABR testing revealed only mild hearing loss on the right side and moderate hearing loss on the left side. This mismatch led to the diagnosis of FHL (Figure 2).

Distortion product otoacoustic emissions (DPOAEs) were evocable bilaterally in five patients. In one case, the test could not be performed due to the patient’s lack of cooperation and the onset of malaise and autonomic symptoms at the start of the examination. In the remaining 10 ears of five patients, OAEs could not be evoked.

Regarding imaging examinations, none of the patients showed signs of central abnormalities on MRI. In two cases, the CT and MRI findings were consistent with known middle ear pathologies.

Psychological assessment and therapy were recommended to all patients; however, only four of them took advantage of this opportunity within the framework of clinical care. All were female, with their ages ranging between 17 and 64. Two out of four had previously had otological diseases (otitis media, otosclerosis), resulting in operations in one case (mastoidectomy, tympanoplasty). Each of them presented with diagnosed or diagnosable psychiatric conditions, such as attention deficit hyperactivity disorder (ADHD; P5), bipolar and substance use disorder (P8), complex post-traumatic stress disorder due to a high number of adverse childhood experiences (cPTSD; P9), and anxiety disorder (P11), and all of them presented a certain degree of alexithymia. The average number of attended sessions was 3.5 (range: 2 to 6), and all patients dropped out prematurely, without proper completion of therapy.

## 4. Discussion

This study aimed to identify FHL in patients with CI presentations at a university clinic over a four-year period. The indications for CI are expanding. However, some patients without objectively severe hearing loss may subjectively perceive their impairment as profound, potentially leading to an inappropriate consideration of CI rehabilitation, particularly when the patient is motivated and open to the procedure. These cases are challenging to recognize and can only be confirmed by a discrepancy between subjective perceptions and objective findings, with the latter indicating higher levels of hearing. Identifying these cases is crucial to ensuring appropriate patient selection and avoiding unnecessary interventions. Given that CI is an expensive, invasive procedure that irreversibly alters the integrity of the cochlea, it is imperative to ensure precise and comprehensive preoperative assessments. Cases of FHL highlight the necessity of utilizing available objective auditory test methods even in situations where additional information from these methods may not be expected, as these tests can serve to confirm or refute subjective measurement results.

Since 2003, our protocol has included mandatory ABR and ASSR examinations for all CI candidates, including adults, even when the indication for implantation appears straightforward. Without this local guideline, the FHL cases described above would have gone unnoticed.

In children, the necessity to determine objective hearing thresholds is obvious. Therefore, several studies highlight the importance of objective hearing assessments, including ASSR and ABR, in childhood [30]. In adults, most recommendations regarding indications for CI focus on pure-tone threshold testing and speech perception tests [3]. The issue of FHL, which is the focus of our study, highlights the importance of objective hearing assessments during the pre-CI evaluation process. ASSR reliably correlates with the actual subjective hearing threshold in both children and adults [21,22,23,26,31]. In our practice, as well as in the international literature, it is widely accepted that ASSR provides a frequency-specific assessment of the actual, objective hearing threshold, and is thus suitable for detecting FHL [32]. 

OAE testing provides valuable information regarding inner ear function [33,34]. However, it is insufficient on its own to confirm or rule out FHL. Our findings demonstrate that OAE responses can sometimes be evoked despite severe hearing impairment, necessitating further evaluation to exclude central organic causes. Conversely, the absence of OAEs do not always correlate with the degree of hearing loss due to varying middle or inner ear damage. Therefore, objective hearing threshold assessment using ASSR and ABR is essential for a comprehensive audiological evaluation.

While most CI candidates present with hearing complaints that clearly justify the surgery, inconsistencies in responses during pure-tone or speech audiometry, such as variations between repeated subjective test results, may indicate FHL. An FHL patient’s ability to communicate with the examiner or relatives may be better than expected based on their hearing thresholds. Even so, there are entirely misleading cases where the patient’s communication appears consistent with their reported hearing loss, even meeting the criteria for CI (for example, P1; details see in Figure 2). These cases demonstrate that identifying a mismatch between PTA and speech recognition alone is not sufficient to detect FHL. Indicators of FHL may be psychological factors identified in the patient’s medical history; however, it is crucial to differentiate between psychological consequences arising from hearing loss and the much rarer FHL. Therefore, in cases where discrepancies persist, an interdisciplinary team including psychologists, psychiatrists, deaf and hard-of-hearing teachers, neurologists, and other allied professionals should be involved.

In the diagnosis of FHL, medical imaging is essential. The aim of the imaging is to rule out intracranial alterations, which can cause central origin hearing loss or psychological abnormalities. MRI is highly recommended to find pathologies of the central nervous system. After the exclusion of organic causes, the psychologist and deaf and hard-of-hearing teacher within our implant team played a significant role recognizing the risk factors and confirming the suspicion of psychological contributors to FHL in the context of functional hearing loss. Furthermore, differentiating FHL from deliberate aggravation or simulation, where patients manipulate their results for personal gain, is also critical. Careful judgment of medical history and revealing tests help to detect such cases.

**Figure 3:**
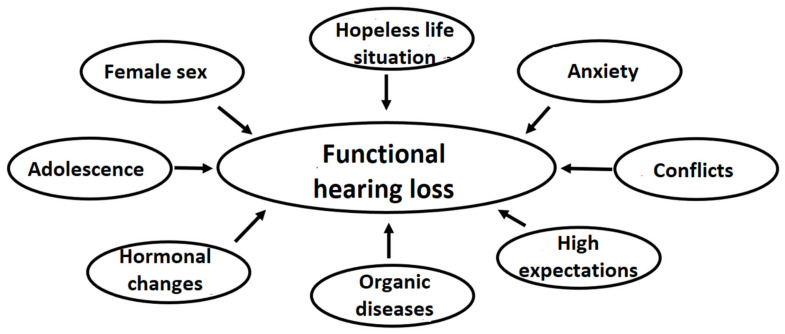
Possible risk factors for functional hearing loss.

The problems underlying functional symptoms can be highly diverse (Figure 3). In adults, functional neurological disorders are mostly associated with family issues, health problems, and workplace difficulties [35]. The female predominance in our patient population aligns with its typical occurrence in other functional diseases, supporting the conclusion that female sex is a risk factor for developing FHL [14,16,19,24,35,36]. In several cases, there was pre-existing organic hearing loss, mostly middle ear problems originated in childhood [37]. Combined with a non-organic overlay. The risk profile of our patients who took up the opportunity to consult with a clinical psychologist included prior or current psychiatric conditions, as described in other studies [38]. All diagnoses found in this study (ADHD, bipolar, substance use, cPTSD and anxiety disorders) have been shown elsewhere to be present in FHL cases [18,39]. 

FHL is a rare condition, particularly at a severity that leads patients to seek CI. Over a four-year period, 11 CI candidates with FHL were identified at our clinic and subsequently excluded from implantation. While this number of cases is insufficient for generalization, the background risk factors observed in these patients align with those reported in the literature on functional disorders. Our findings highlight the importance of objective auditory testing and multidisciplinary teamwork in accurately identifying FHL. Avoiding unnecessary surgeries is crucial for both patients and the efficient use of healthcare resources. Although objective testing comes with a considerable cost, this does not compare to the financial burden of a CI, especially if a patient disappointed by a noneffective CI later applies for a removal. Thus, the advantages of objective measurement clearly outweigh its disadvantages. Other studies have also reported similar cases [40,41,42] and concluded that treating these patients with CI would not only be ineffective and unnecessarily risky, but also lower the chance of full recovery [43]. 

Many patients did not accept the diagnosis of FHL, and either dropped out of psychotherapy or refused it altogether. Their medical histories and medium-length follow-ups indicate that these patients tended to turn to other institutions for further examinations. The low success rate of psychotherapy is not unfamiliar to therapists working with functional symptom disorder patients. Psychotherapists often describe the management of affected patients as challenging and somewhat frustrating, mainly due to the difficult diagnostic process and the “somatic fixation” of some patients [36]. Also, these patients often come through referral, and thus are reluctant to attend, although higher acceptance of, and motivation for, psychotherapy have been found to positively influence long-term treatment outcomes in somatization patients [44]. Some of them, despite clear communication on their therapeutic goals, may have hidden expectations that they will finally receive a cochlear implant if they attend the sessions, and become disappointed when they find out that this will not be the case. All of this calls for accurate, but sensitive, nonconfrontational communication during doctor referrals and initial psychotherapy sessions [45]. 

## 5. Conclusions

The diagnostic process of FHL is very difficult, requiring a complex series of tests and interdisciplinary collaboration. Without a correct diagnosis, patients may receive inappropriate treatment, hearing aids, or even CI. Based on our experience, we strongly emphasize the necessity of objective hearing loss measurements such as ABR and ASSR in all cases considered for CI. These examinations should be performed even when communication difficulties, environmental assessment, and the patient’s motivation would clearly support an indication for CI.

## Figures and Tables

**Table 1:** Demographic data and summary of audiological assessment results for each patient. Pure-tone audiometry (PTA) and auditory steady-state response (ASSR) values measured at four frequencies (500, 1000, 2000, and 4000 Hz) were averaged separately for the right and left ears. Functional hearing loss (FHL) values were calculated as the difference between the PTA and ASSR thresholds. The distortion product otoacoustic emission (DPOAE) results are presented as evocable (+) or non-evocable (−) for both the right and left ears. The identified organic causes of hearing loss and observations regarding patient communication during the examination are also presented.

Patient	Age (Years) Female/Male	PTA (dB)		ASSR (dB)		FHL (dB)		DPOAEs	Organic Background	Communication During Examination
		Right Ear	Left Ear	Right Ear	Left Ear	Right Ear	Left Ear	R/L		
P1	32 M	110	120	34	46	76	74	−/−	yes (inner ear)	no communication
P2	37 F	120	* 75	52.5	45	67.5	30 *	−/−	yes (otosclerosis)	minimal difficulty
P3	13 F	97.5	100	36	44	61.5	56	−/−	yes (middle ear)	minimal difficulty
P4	41 F	106	90	46	52.5	60	37.5	−/−	yes (inner ear)	minimal difficulty
P5	17 F	77.5	77.5	19	11	58.5	66.5	+/+	no	proper communication
P6	22 F	100	100	26	24	74	76	+/+	no	minimal difficulty
P7	15 F	120	120	37.5	25	82.5	95	intolerance	no	minimal difficulty
P8	64 F	* 71	91	37.5	59	33.5 *	32	−/−	yes (inner ear)	difficulty
P9	54 F	109	92.5	39	50	70	42.5	+/+	yes (inner ear)	difficulty
P10	15 F	95	95	26	25	69	70	+/+	no	no communication
P11	33 F	95	* 76	47.5	72.5	47.5	3.5 *	+/+	yes (inner ear)	proper communication

* No functional hearing loss or not a candidate for CI.

## Data Availability

The original contributions presented in this study are included in the article. Further inquiries can be directed to the corresponding author.

## Abbreviations

ABR	auditory brainstem response
ADHD	attention deficit hyperactivity disorder
ASSR	auditory steady-state response
CI	cochlear implantation
cPTSD	complex post-traumatic stress disorder
FHL	functional hearing loss
HRCT	high-resolution computed tomography
MRI	magnetic resonance imaging
OAE	otoacoustic emission
PCCT	photon counting computed tomography
PTA	pure-tone audiometry
